# Supplementary material for: The psychotomimetic ketamine disrupts the transfer of late sensory information in the corticothalamic network
Source: Eur J Neurosci. 2022 Nov 1;57(3):440–55. doi: 10.1111/ejn.15845 (PMC10092610; doi:10.1111/ejn.15845)
Supplement: Supplementary file 1 — Figure S1: Ketamine does not change the multi‐scale entropy in the beta band between the cortex and thalamus. Comparison of the multi‐scales entropies of saline (blue) and ketamine (red) conditions at all recording sites in the beta band during the 200‐700 ms post‐stimulus period. Layer 6 and VPm show a significant increase in entropy. Each scale point is the average entropy (± SEM, from 40 values, 10 per rats, 4 rats). The statistical test does not reveal any significant difference between the saline and ketamine conditions (p > 0.05, paired t‐test). [file EJN-57-440-s001.pdf]

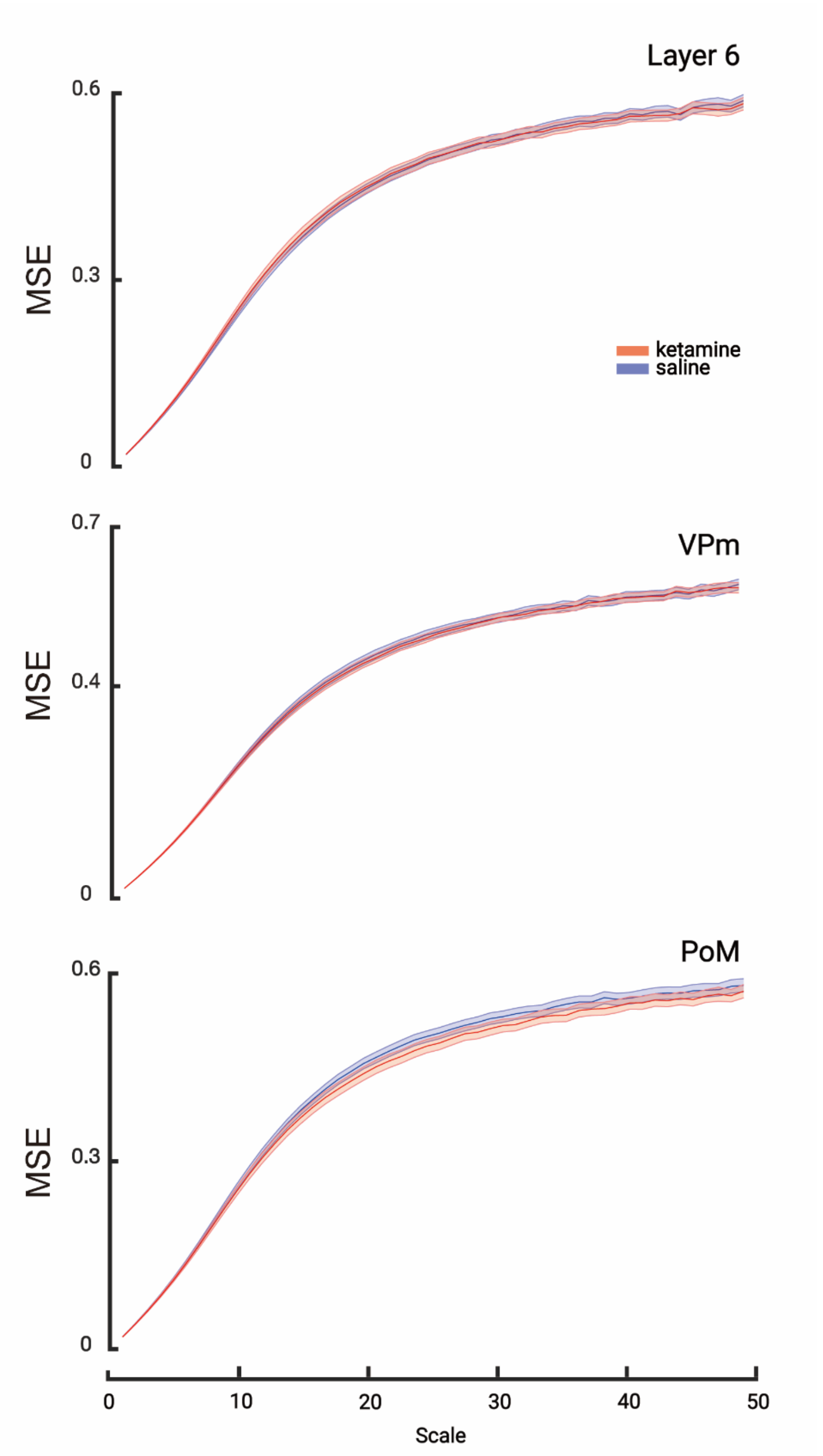

Figure S1: Ketamine does not change the multi-scale entropy in the beta band between the cortex and thalamus. Comparison of the multi-scales entropies of saline (blue) and ketamine (red) conditions at all recording sites in the beta band during the 200-700 ms post-stimulus period. Layer 6 and VPm show a significant increase in entropy. Each scale point is the average entropy (± SEM, from 40 values, 10 per rats, 4 rats). The statistical test does not reveal any significant difference between the saline and ketamine conditions ($p > 0.05$, paired t-test).
